# Mammary tuberculosis – importance of recognition and differentiation from that of a breast malignancy: report of three cases and review of the literature

**DOI:** 10.1186/1477-7819-5-67

**Published:** 2007-06-18

**Authors:** Müfide Nuran Akçay, Leyla Sağlam, Pınar Polat, Fazlı Erdoğan, Yavuz Albayrak, Stephen P Povoskı

**Affiliations:** 1 Department of General Surgery, Atatürk University Medical Faculty, Erzurum, Turkey; 2 Department of Chest Diseases, Atatürk University Medical Faculty, Erzurum, Turkey; 3 Department of Radiology, Atatürk University Medical Faculty, Erzurum, Turkey; 4 Department of Pathology, Atatürk University Medical Faculty, Erzurum, Turkey; 5 Department of Division of Surgical Oncology, Department of Surgery, The Arthur G. James Cancer Hospital and Richard J. Solove Research Institute and Comprehensive Cancer Center, The Ohio State University, Columbus, Ohio, 43210, USA

## Abstract

**Background:**

While tuberculosis of the breast is an extremely uncommon entity seen in western populations, it accounts for up to 3% of all treatable breast lesions in developing countries.

**Case presentations:**

We reviewed three female cases of mammary tuberculosis that were diagnosed and treated in Turkey during the same calendar year. All three patients presented with a painful breast mass. In all cases, fine needle aspiration was nondiagnostic for mammary tuberculosis. However, the diagnosis of mammary tuberculosis was confirmed by histopathologic evaluation at the time of open surgical biopsy. All three patients were treated with antituberculous therapy for six months. At the end of the treatment period, each patient appeared to be clinically and radiologically without evidence of residual disease.

**Conclusion:**

The diagnosis of mammary tuberculosis rests on the appropriate clinical suspicion and the histopathologic findings of the breast lesion. Its recognition and differentiation from that of a breast malignancy is absolutely necessary. Antituberculous chemotherapy, initiated immediately upon diagnosis, forms the mainstay of treatment for mammary tuberculosis.

## Background

Despite the fact that more than 8.9 million people are diagnosed annually with tuberculosis worldwide [1], isolated tuberculosis of the breast is an extremely uncommon entity. While mammary tuberculosis is globally reported to account for less than 0.1% of all known breast diseases, it is reported in developing countries to comprise up to 3% of treatable breast lesions [2-5]. In the current report, we have reviewed three cases of mammary tuberculosis that were diagnosed and treated within three Turkish female patients within the same calendar year.

## Case presentations

### Case 1

**Figure 1:**
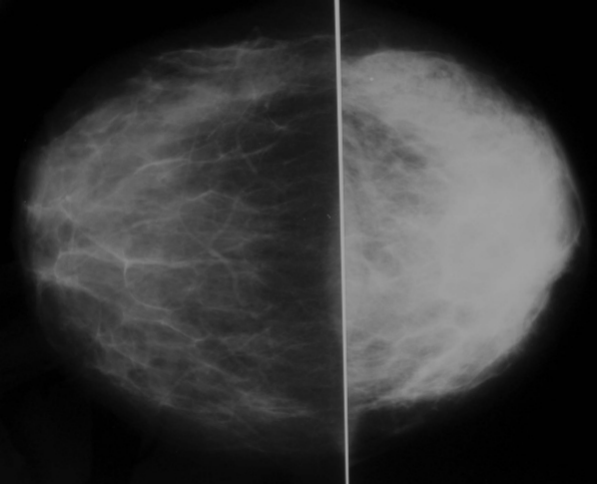
Craniocaudad mammographic views showing generalized increased radioopacity within the left breast.

A 45 year-old female patient presented with a 15 day history of a painful left breast mass, an associated sinus tract, and low-grade fever. She had received empiric antibiotic (antibacterial) therapy during this period. However, her symptoms had not resolve. She had no family history of breast cancer. Physical examination revealed a tender, erythematous left breast mass with an associated sinus tract. There was no clinically palpable left axillary lymphadenopathy. On left breast ultrasound, there was an ill-defined, hypoechoic, heterogenous, 3 cm lesion in the upper-inner quadrant of the left breast. Additionally, ultrasound revealed a 5 mm sinus tract connecting the breast lesion to the skin and a 4 mm focal ductal dilatation in the upper-outer quadrant of the same breast. Mammography showed generalized increased radioopacity throughout the left breast (Figure 1). Fine needle aspiration (FNA) revealed only inflammatory cells, including neutrophils, lymphocytes, and abundant macrophages. Therefore, the left breast mass was completely excised by an open surgical biopsy. The histopathologic evaluation of the specimen revealed granulomas with central caseaous necrosis, epitheloid histiocytes, Langhans' giant cells, and an intense lymphocytic infiltration at the periphery of the granulomas. The patient was treated with antituberculous therapy, consisting of rifampicin (450 mg per day), isoniazid (300 mg per day), pyrazinamide (1500 mg per day), and ethambutol (800 mg per day) for two months and was followed by rifampicin and isoniazid for an additional four months. At the end of the six-month antituberculous treatment period, the patient appeared to be clinically and radiologically without evidence of residual disease.

### Case 2

**Figure 2:**
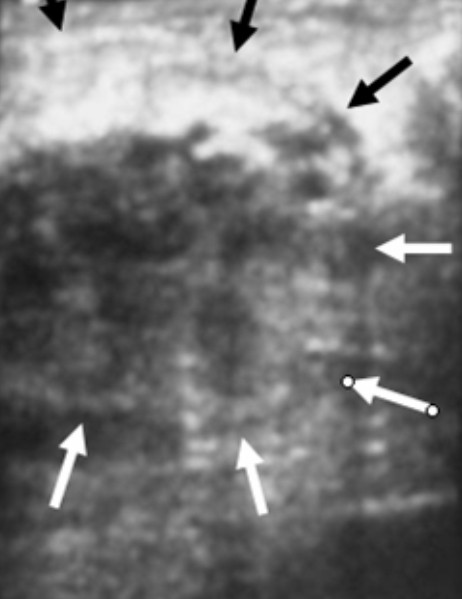
An ill-defined, hypoechoic, heterogenous 6 cm lesion is seen in the upper-inner quadrant of the left breast on ultrasonography.

**Figure 3:**
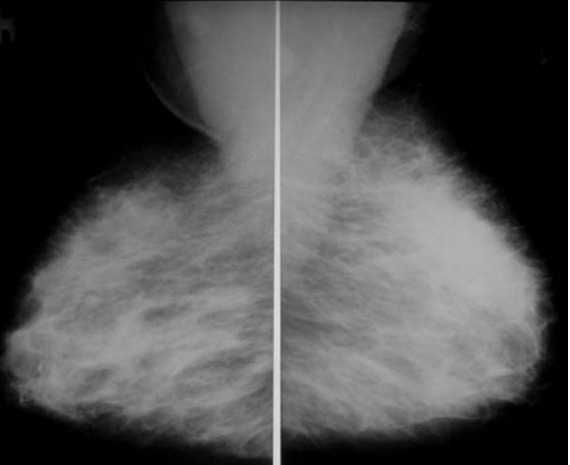
Mediolateral oblique mammographic views showing increased radioopacity within the upper pole of the left breast.

A 39 year-old female patient presented with a chronic four year history of a painful left breast mass, an associated sinus tract, painful left axillary lymphadenopathy, and low-grade fevers. She had received empiric antibiotic (antibacterial) therapy and had previously undergone drainage of the breast lesion on several occasions, but with no relief of her symptoms. She had no family history of breast cancer. Physical examination revealed a tender, erythematous left breast mass with an associated sinus tract located in the upper-inner quadrant of the left breast. This was associated with a 2 cm firm area of tender adenopathy within her left axilla. Left breast ultrasound revealed an ill defined, hypoechoic, heterogenous lesion, measuring 6 cm in size and located in the upper-inner quadrant of the left breast (Figure 2) and two enlarged left axillary lymph nodes (measuring 24 × 21 mm and 15 × 11 mm) with associated microcalcification. Mammography revealed increased radioopacity within the upper pole of the left breast (Figure 3). An FNA was performed and revealed that the left breast mass was filled with purulent material. Therefore, this presumed breast abscess was surgically managed with an incision and drainage procedure, with random biopsies taken from the wall of the abscess cavity. Histopathological evaluation of the specimen confirmed the diagnosis of mammary tuberculosis. The patient was treated with the same antituberculous drug regimen for six months, as outlined above. At the end of the antituberculous treatment period, the patient appeared to be clinically and radiologically without evidence of residual disease, including resolution of her left axillary adenopathy.

### Case 3

**Figure 4:**
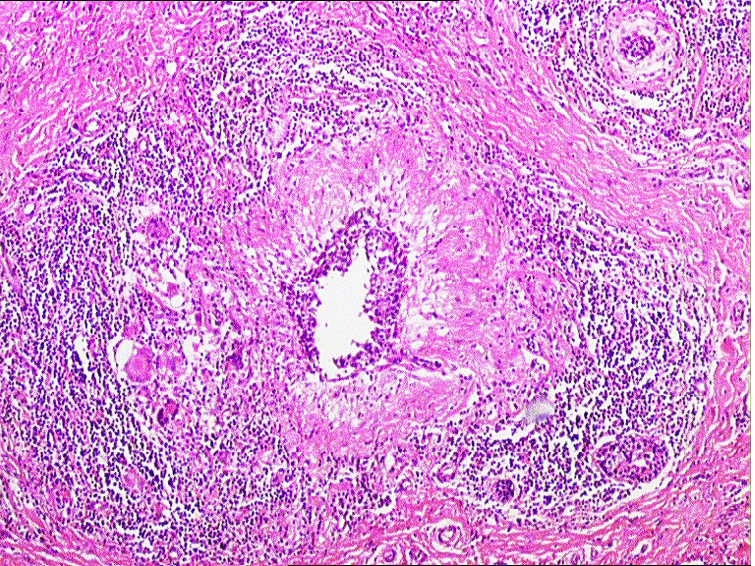
The histopathologic examination of the specimen revealed granulomas with central caseation necrosis, epitheloid histiocytes, Langhans' giant cells, and intense lymphocytic infiltration at the periphery of the granulomas (H&E, ×40).

A 30 year-old female patient was hospitalized with the complaints of a four month history of a painful right breast mass and low-grade fever. During that same time period, she had been evaluated by several other physicians and had previously received empiric antibiotic (antibacterial) therapy for mastitis, but with no relief of her symptoms. She had no family history of breast cancer. Physical examination revealed a tender, erythematous, firm right breast mass without an associated sinus tract within the upper inner quadrant of her right breast. She had no clinically palpable right axillary lymphadenopathy. A right breast ultrasound reported finding consistent with a breast abscess. FNA revealed only inflammatory cells. Therefore, the right breast mass was completely excised by an open surgical biopsy. The histopathological evaluation of the specimen revealed findings consistent with mammary tuberculosis (Figure 4). This patient was also treated with the same antituberculous chemotherapy for six months. At the end of the antituberculous treatment period, the patient appeared to be clinically and radiologically without evidence of residual disease.

In all three patients, routine hematologic and biochemical parameters were normal, including negative testing for HIV. The third patient did have a marginally elevated erythrocyte sedimantation rate of 20 mm/hour (normal range: below 20 mm/hour). Plain-film chest radiography and high resolution computed tomography were normal in all three cases. The diameter of tuberculin skin test (Mantoux) was greater than 15 mm at the end of 72 hour in all three patients. Finally, in all three cases, the surgical specimens proved negative for acid-fast bacilli on staining, as well as negative on culture (Lowenstein Jensen). Due to various technical constraints, the mycobacteriology laboratory at Atatürk University Medical Facility in Erzurum, Turkey did not have the capability of performing polymerase chain reaction (PCR) for the identification of tuberculosis at the time of the evaluation of these three patients.

## Discussion

Mammary tuberculosis is an extremely uncommon disease entity, especially in western populations [2-5]. In this regard, it has been postulated that mammary gland tissue, like that of the spleen and skeletal muscle, may convey some degree of resistance to the survival and propagation of the tubercle bacillus organism [3].

Mammary tuberculosis may be primary, when no demonstrable tuberculous focus exists elsewhere in the body, or secondary to a pre-existing lesion located elsewhere in the body. Primary tuberculous infection of the breast may occur through skin abrasions or through the milk duct openings on the nipple [4,6]. Direct extension from contiguous structures like the underlying ribs is another possible mode of infection, as previously reported by Eroğlu *et al*., [7]. However, it is generally believed that tuberculous infection of the breast is usually secondary to a pre-existing tuberculous focus located elsewhere in the body. Such a pre-existing focus could be of pulmonary origin or could be a lymph node within the paratracheal, internal mammary, or axillary nodal basins. Involvement of the breast in such cases of secondary tuberculous infection is presumed to be by direct hematogenous spread [4,6]. Although the diagnosis of primary mammary tuberculosis generally requires positive identification of acid-fast bacilli and/or positive cultures, two of our patients (case 1 and case 3) were presumed to be examples of primary mammary tuberculosis, because another tuberculous focus could not be found. However, the second patient (case 2) had an axillary lymph node associated with the breast lesion. In this particular case, she was presumed to have a secondary tuberculous infection.

Although mammary tuberculosis is much more common in females, it has been previously reported to also occur in males [2,4,5]. In the current case report, all three patients were females. Likewise, Lilleng *et al*., [8], in a study of 809 cases of male breast mass, did not report finding a single case of mammary tuberculosis. Nevertheless, Khanna *et al*., [2] reported two cases of male mammary tuberculosis within a series of 52 patients, Shinde *et al*., [4] reported three cases of male mammary tuberculosis within a series of 100 patients, and Harris *et al*., [5] reported one case of male mammary tuberculosis within a series of 38 patients.

The clinical presentation of mammary tuberculosis is somewhat variable [2-6,9,10]. Constitutional symptoms of tuberculosis (fever, weight loss, night sweats, or failing of general health) are infrequently encountered. In the series by Khanna *et al*., [2], Shinde *et al*., [4], and Harris *et al*., [5], 21%, 20%, and 16% of patients, respectively, had such constitutional complaints. In our current series, all three patients did report low-grade fevers. In the series by Khanna *et al*., [2], they reported the finding of a breast mass with an associated sinus tract in 39% of cases, an isolated breast mass in 23%, a sinus tract without a breast mass in 12%, and tender breast nodularity in 23%. In the series by Harris *et al*., [5], they reported the finding of an isolated breast mass in 52% of cases, a breast mass with an associated sinus tract in 34%, multiple sinus tracts without a breast mass or sinus tracts from previously drained breast abscesses in 23%, and tender breast nodularity in 13%. Associated axillary lympadenopathy has been reported in 40% to 71% of affected individuals [2,5,9,10]. An isolated breast mass without an associated sinus tract can commonly mimic the presentation of a breast cancer, since the clinically palpable breast mass is usually firm, ill-defined, irregular, and can be associated with fixation to the skin [2,4,5]. This diagnostic dilemma can be further complicated by the presence of associated axillary adenopathy. However, pain and palpable tenderness is associated far more frequently with a tuberculous mass than with a malignant breast mass, and involvement of the nipple and areola complex is less commonly seen in mammary tuberculosis [2,4,5].

In our particular series, confusion with that of the diagnosis of a breast cancer could have been entertained since all three patients had a firm, irregular breast mass with varying degrees of fixation to the overlying skin and since the second patient presented with associated axillary lympadenopathy. Therefore, histopathologic evaluation at the time of open surgical biopsy was critical to confirming the diagnosis of mammary tuberculosis and to excluding the diagnosis of breast cancer. The question as to whether or not core (Tru-cut^(R)^) biopsy of these tuberulous breast masses could have as effectively made the correct diagnosis as open surgical biopsy can not be answered by the currect report.

Another disease process that must be kept in mind in the differential diagnosis of mammary tuberculosis is that of idiopathic granulomatous mastitis (GM). GM is an uncommon breast lesion that was first described by Kessler and Woolloch [11]. The etiology of most cases is idiopathic, and should be distinguished from other rare granulomatous conditions, including tuberculosis, sarcoidosis, and Wegener's granulomatosis. Idiopathic GM usually occurs in women of reproductive age, and may be associated with lactation or may occur in the postpartum period. In both idiopathic GM and mammary tuberculosis, there is a usually a hard breast lump in the clinical presentation. The accurate diagnosis of idiopathic GM versus that of mammary tuberculosis is based upon the particular histological features of the biopsy specimens. In mammary tuberculosis, the most common features include caseous necrosis, epitheloid histiocytes, Langhans' giant cells, and granulomas. The presence of predominantly neutrophils in the background, and the lack of caseous necrosis may favor a diagnosis of idiopathic GM rather than that of mammary tuberculosis [12,13]. In all of our cases, the presence of caseous necrosis, in addition to other features mentioned above in the histologic evaluation of biopsy specimens, favored the diagnosis of mammary tuberculosis.

Lastly, any patient presenting with a breast mass that is associated with a draining sinus tract needs to be differentiated from actinomycosis by the absence of sulfur granules in the discharge and by fungal culture [2].

Breast imaging modalities, such as mammography and ultrasound, may be useful adjuvant diagnostic tools in the overall process of diagnosing mammary tuberculosis [10]. However, these imaging modalities are by no means reliable in distinguishing mammary tuberculosis from that of a breast malignancy [2,4,5,10]. The most common mammographic findings are dense breast parenchyma with or without an associated ill-defined mass-like density and associated skin thickening. In the study by Sakr *et al*., [10], mammography revealed a breast mass mimicking a malignant tumor is 30% of cases, identified axillary or intramammary adenopathy in 40% of cases, and identified skin thickening and nipple retraction in 20% of cases. Likewise, in the study by Sakr *et al*., [10], breast ultrasound revealed a suspicious hypoechoic mass in 60% of cases and axillary adenopathy in 50% of cases. Obviously, all of these findings are nonspecific and nondiagnostic for the diagnosis of mammary tuberculosis. Conversely, Makanjuola *et al*., [14], reported that the mammographic demonstration of a dense sinus tract connecting an ill-defined breast mass to an area of localized skin thickening is strongly suggestive of a tuberculous breast abscess. However, such a finding is found in only a small percentage of patients [14]. In our series, ultrasound revealed a 5 mm sinus tract connecting the breast lesion to the skin for the first case and 6 cm heterogenous breast mass and two suspicious axillary lymph nodes for the second case. However, in all three cases, mammography showed increased radioopacity within the breast tissue, which was nonspecific and nondiagnosis.

The accurate diagnosis of mammary tuberculosis has traditionally relied upon the demonstration of a classical caseous lesion, acid-fast bacilli within such a lesion, and/or the demonstration of epitheloid granulomas, Langhans' giant cells, and lymphocytic aggregates [2,7]. It has been more recently recognized that an AFB-positive smear is not always sufficient evidence for the definitive mycobacterial diagnosis of tuberculosis. Differentiation of *Mycobacterium tuberculosis *from other *Mycobacteria species *represents an important health issue. Technically advanced mycobacteriology laboratories rely on cutting edge methology, particularly DNA-RNA hibridization with a culture grown in BACTEC 7H12 or NAP test with the same culture. Other advanced methods, such as High Performance Liquid Chromatography (HPLC), Gas Liquid Chromatography (GLC), nucleic asid probes, and PCR, are also used for identification of *Mycobacterium tuberculosis *(15). These methods are all commonly utilized in technically advanced mycobacteriology laboratories. However, such advanced mycobacteriology laboratory capabilites are not currently available at the Atatürk University Medical Facility in Erzurum, Turkey. Therefore, we primarily use staining, smear examination, and culture in our mycobacteriology laboratory for the diagnosis of tuberculosis. In our mycobacteriology laboratory, smear and culture positivity were founded as 46 % and 63 %, respectively, as recently published by Saglam *et al*., [16] from our institution. FNA is generally a reliable diagnostic procedure, particularly if the aspirated material can be examined by the stains for acid fast bacilli [5,17]. In our present report, FNA was performed but was not diagnostic for mammary tuberculosis. The accurate diagnosis was only achieved after histopathologic evaluation of the open surgical biopsy specimens.

Medical treatment with a four drug regimen of rifampicin, isoniazid, pyrazinamide, and ethambutol forms the basis of treatment of mammary tuberculosis [2,4,5]. Surgical intervention is generally reserved for diagnostic purposes, aspiration/drainage of a residual breast mass (representing a "cold abscess"), excision of a residual mass, and excision of a residual sinus tract. In refractory cases of mammary tuberculosis causing significant destruction of the breast tissue, simple mastectomy may need to be considered [2,4]. In two of our reported cases, we completely excised the breast mass by an open surgical biopsy. In the other case, only an incision and drainage procedure and incisional biopsy was performed. At the end of the six-month antituberculous therapy period, all three patient appeared to be clinically and radiologically without evidence of residual disease.

## Conclusion

Mammary tuberculois should be considered in the differential diagnosis of any case of a painful breast mass, mastitis, or breast abscess that appears refractory to conventional therapy. Its recognition and differentiation from that of a breast malignancy is absolutely necessary. The diagnosis rests on clinical suspicion and histopathologic findings. Antituberculous chemotherapy, initiated immediately upon diagnosis, forms the mainstay of treatment for mammary tuberculosis.

## Competing interests

The author(s) declare that they have no competing interests.

## Authors' contributions

**MNA **performed all the procedures on the patients, and conceived, designed and coordinated the writing of the manuscript.

**LS **ordered the medical therapy of the patients, and participated in the writing of the manuscript.

**PP **carried out the radiologic studies, and participated in the writing of the manuscript.

**FE **carried out the histopathology studies.

**YA **participated in the design of the study and the writing of the manuscript.

**SPP **participated in the writing and translation of the manuscript.

All authors read and approved the final version of the manuscript.
